# Application of Ethylcellulose Coating to Hydrophilic Matrices: A Strategy to Modulate Drug Release Profile and Reduce Drug Release Variability

**DOI:** 10.1208/s12249-014-0128-5

**Published:** 2014-05-22

**Authors:** Raxit Y. Mehta, Shahrzad Missaghi, Sandip B. Tiwari, Ali R. Rajabi-Siahboomi

**Affiliations:** Global Headquarters, Colorcon, Inc., 275 Ruth Rd., Harleysville, Pennsylvania 19438 USA

**Keywords:** bio-relevant media, drug release variability, ethylcellulose coating, hydrophilic matrix tablets, hypromellose

## Abstract

Hydrophilic matrix tablets are commonly used for extended release dosage forms. For low aqueous-solubility drugs, there may be challenges in modulation of release profiles and achieving consistent release in physiological conditions. To evaluate potential formulation strategies, matrix tablets of a low-soluble drug, hydrochlorothiazide, were developed using hypromellose and two fillers of different solubility, lactose (soluble) or partially pregelatinized maize starch (partially soluble). Additionally, application of an insoluble barrier membrane, aqueous ethylcellulose coating system, and a hydrophilic pore former onto matrix tablets was evaluated. Drug release from uncoated matrix tablets was variable at different agitation rates. Evaluation of tablets in bio-relevant media using physiologically relevant residence time indicated variable and higher initial release rate for uncoated matrices containing lactose but more robust behavior for tablets containing partially pregelatinized starch. Such *in vitro* behavior may lead to erratic drug release *in vivo*, when comparing fed *versus* fasted conditions. Dissolution profiles from barrier membrane-coated tablets showed initial delay, followed by zero-order release kinetics, with reduction or elimination of variability compared to uncoated matrices. Such reduced variability may mitigate mechanical effects of post-prandial stomach. Effects of coating weight gain and inclusion levels of pore former were evaluated and found to be critical in achieving robust and stable release profiles.

**Electronic supplementary material:**

The online version of this article (doi:10.1208/s12249-014-0128-5) contains supplementary material, which is available to authorized users.

## INTRODUCTION

Hydrophilic matrix technology has been widely used for oral controlled delivery of various drugs. The advantages of this technology are ease of formulation, cost-effective manufacturing process, wide regulatory acceptance of the polymer systems, and flexibility in the control of the drug release profiles. Hypromellose (hydroxypropyl methyl cellulose, HPMC) is the most commonly used polymer in formulation of extended release (ER) hydrophilic matrix tablets. When a hydrophilic matrix dosage form is exposed to the gastrointestinal (GI) fluid, the polymer on the surface of the dosage form hydrates and swells, forming a protective gel layer from which the drug is gradually and continuously released over time, either by diffusion through the polymeric gel layer, by erosion of the gel layer, or by a combination of these mechanisms (1,2).

The key to optimal formulation design for hydrophilic matrix systems is the formation of a robust gel structure which enables consistent drug release, irrespective of the changes in the GI tract. The major factors governing formation and strength of the gel layer are the chemistry, viscosity grade, concentration, and particle size of the hydrophilic polymer. In the case of a low aqueous-solubility drug, where the drug release is predominantly controlled by erosion of the polymeric gel layer, low viscosity grades of hypromellose (e.g., METHOCEL™ K100LV premium cellulose ethers) are generally recommended to achieve complete and consistent drug release from matrix tablets. Low inclusion level of such grades of hydrophilic polymers, however, may contribute to lower gel strength (3) and further result in variable and inconsistent release for some drugs. This effect could be critical when matrix tablets are exposed to the post-prandial stomach. The physiological environment of the stomach (i.e., presence of food, mechanical forces, location of matrix tablets within or on the surface of stomach content, availability of the hydrating liquid, effect of acid secretion, and residence time) may alter the motility and transit time of the dosage forms through the GI tract. As a result of the peristaltic action of the stomach, matrix tablets could be subjected to intensified shear forces for an extended period of time, which may change the erosion pattern of the hydrophilic polymer and lead to an undesirable increase in drug release. For matrix tablets of low-soluble drugs, it is important to accurately predict such effects and to modulate and achieve consistent release using the compendial dissolution testing. In some cases the variation in the agitation rate may be a useful approach to simulate the excessive mechanical stress observed in the fed state (4–7). In addition, the change of pH and/or ionic strength of the media as well as the use of fed and fasted simulated media (FaSSIF and FeSSIF) can provide better insight as to the fate of the hydrophilic matrix tablets in the GI tract (8–13).

The initial approach to achieve a robust matrix system is to use an optimal concentration of the rate controlling polymer and obtain consistent gel formation. Moreover, inclusion of excipients, such as partially pregelatinized maize starch (Starch 1500®) can enhance the strength of the gel layer and prevent premature erosion of the hydrated matrix tablets (14). In addition, the application of an insoluble barrier membrane coating over hydrophilic matrices has been utilized as an approach to modulate drug release from matrix tablets by attaining zero-order drug release kinetics (15). The presence of a barrier membrane coating may also provide protection against shear forces observed in the post-prandial stomach and offer consistent drug release throughout the GI tract.

To further explore the utility of this approach, the objective of the present study was to investigate the application of a barrier membrane coating, containing ethylcellulose, and a pore former, onto matrix tablets of a very low-soluble model drug, hydrochlorothiazide. The combination of barrier membrane and hydrophilic matrix system was utilized as a strategy to modulate drug release from hydrophilic matrices and to reduce the overall variability in release. Such variability was associated with the difference in drug release upon changing the agitation rate within the dissolution media. The influence of coating weight gain and pore former level within the coating was also investigated. In conjunction with the coating, the effect of filler type within the core formulation of matrix tablets was also evaluated. Furthermore, in order to evaluate the performance of the ethylcellulose coated matrices over time, the tablets were subjected to stability testing under different ICH storage conditions.

## MATERIALS AND METHODS

### Tablet Formulation and Manufacture

**Fig. 1:**
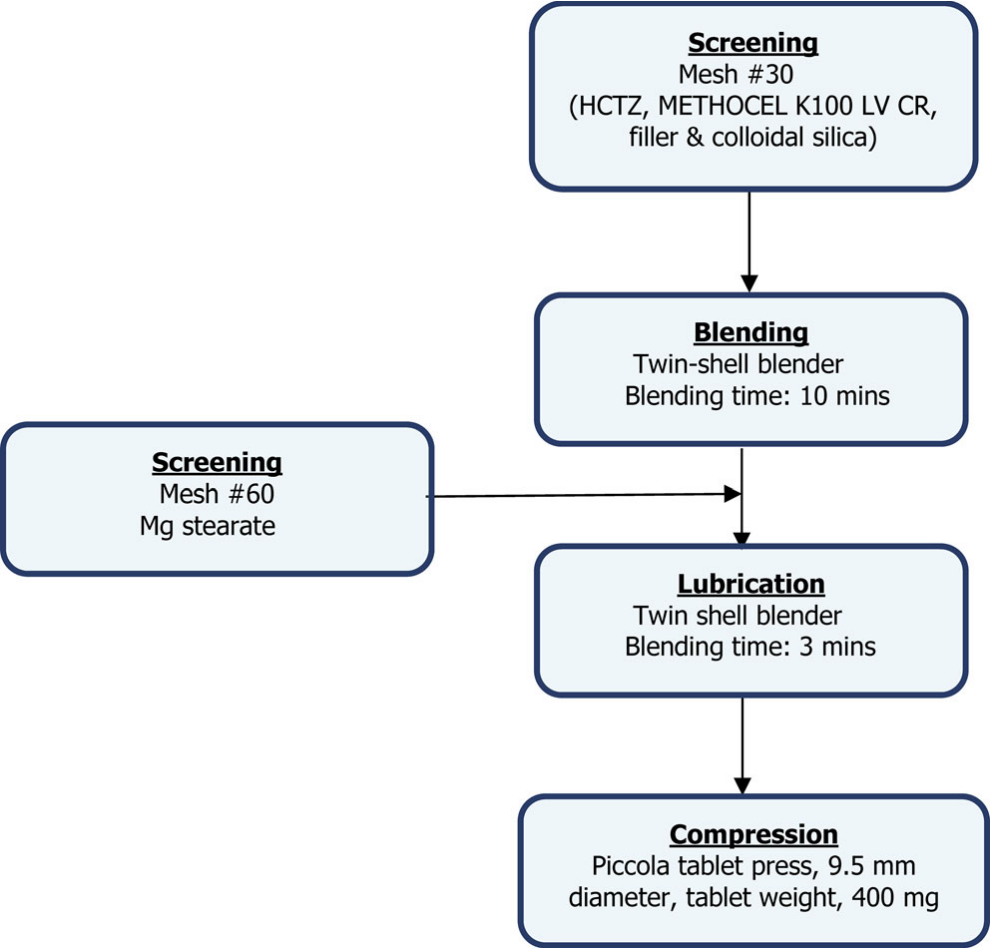
Process flow chart for production of HCTZ matrix tablets

In this study, hydrochlorothiazide (HCTZ), a very slightly water-soluble diuretic compound (∼0.7 mg/ml) (16) was used as a model drug at the dose level of 200 mg. The composition of hydrochlorothiazide ER matrix tablets is shown in Table I. HCTZ, HPMC (METHOCEL K100LV Premium CR), filler (Starch 1500 or lactose) and colloidal silicon dioxide were sieved through a screen, US-standard mesh #30 (600 μm), added to a twin-shell blender (Patterson-Kelley, USA), and mixed for 10 min. The powder blends were then lubricated with magnesium stearate for 3 min and compressed on an instrumented rotary tablet press (Piccola, Riva, Argentina) using standard concave tooling (9.5 mm) at a target tablet weight of 400 mg (Fig. 1). Matrices with tablet hardness greater than 15 kp (3.4 MPa) were used for application of ethylcellulose coating as an insoluble barrier membrane.

**Table I:** Composition of Extended Release Hydrochlorothiazide Matrix Tablets

Ingredients	Supplier	Composition (% *w/w*)	
Hydrochlorothiazide (HCTZ)	Hubei Maxpharm, China	50.0	50.0
Hypromellose (METHOCEL™ K100LV Premium CR)	The Dow Chemical Company, USA	30.0	30.0
Lactose monohydrate (FastFlo)	Foremost, USA	–	19.0
Partially pregelatinized starch (Starch 1500)	Colorcon, USA	19.0	–
Colloidal silica (CAB-O-Sil® M5P)	Cabot Corp., USA	0.5	0.5
Magnesium stearate	Mallinckrodt, USA	0.5	0.5
Total		100.0	100.0

### Application of Ethylcellulose Coating

Matrix tablets of HCTZ were coated with an insoluble barrier membrane (BM) using aqueous ethylcellulose coating, Surelease® (containing medium chain triglyceride as plasticizer) and an HPMC-based Opadry® (HPMC low viscosity grade, 6 cps, triacetin, and talc) as a pore former, at 85:15 and 60:40 *w/w* ratios. The ratio of Surelease to Opadry was calculated based on total dry solids. Prior to application, the coating systems were dispersed in water at 10% *w/w* solids content. Tablets (batch size, 1 kg) were then coated to 2%–8% *w/w* weight gain (WG) in a fully perforated coating pan (LABCOAT I, O’Hara Technologies, Canada) using a 1-mm nozzle (970/7-1S75, Schlick, Germany). Standard coating processing parameters were used for application of aqueous ethylcellulose barrier membrane coating (i.e., product temperature, 42°C–45°C; spray rate of 6–8 g/min; air flow of 290 m^3^/h).

It is generally known that organic application of ethylcellulose coating can result in stronger film compared to the aqueous system. For comparison, the HCTZ matrix tablet cores containing lactose were also coated using a combination of ethylcellulose (ETHOCEL™ Standard 20 Premium) and hypromellose (METHOCEL E5LV) (The Dow Chemical Company, USA), as a pore former, at 85:15 and 60:40 *w/w* in a solvent mixture of isopropanol and water, 90:10 *w/w*, at 7% solids content.

### Drug Release Study

The uncoated and ethylcellulose coated matrix tablets were subjected to dissolution testing in different media (*n* = 3–6), as outlined below to mimic fed and fasted conditions and compare the dissolution performance of the tablets. The drug release profiles were compared using similarity factors (*f*_*2*_), where *f*_*2*_ values of 50 to 100 indicate similarity in drug release between the evaluated tablets (23).

#### *Compendial Media*

Drug release testing of HCTZ matrix tablets were conducted using USP apparatus II, paddles, (Agilent Technologies, Cary, USA) with sinkers and 900 ml of dissolution media at 37°C ± 0.5°C. The fed state was simulated using a two-stage dissolution method. The tablets were first exposed to acetate buffer, pH 4.5, for 4 h, followed by phosphate buffer, pH 6.8, (at 100 rpm) for the remainder of the test (12 h). The elevated mechanical stress observed in the fed stomach was simulated by varying the agitation rates during the first stage of dissolution testing at 50, 100, and 150 rpm. The HCTZ release was determined using a UV/Vis spectrophotometer (Cary 50, Agilent technologies, USA) at a wavelength of 272 nm.

#### *Bio-Relevant Media*

Dissolution testing was performed using USP apparatus III, reciprocating cylinder, (BIO-DIS, Agilent Technologies, Cary, USA) at 37°C. The bio-relevant dissolution media, FaSSIF phosphate buffer, FeSSIF acetate buffer, FaSSIF, and FeSSIF were prepared according to Phares SIF Powder Preparation Protocol. Ensure Plus was used directly as purchased with no modification (9,10). The reciprocating cylinder was operated at 10 dips per minute (dpm) with multiple steps of media change over to simulate the human gastrointestinal environment. The media loading sequence for USP apparatus III is described in Tables II and III (11). All samples were diluted at 1:10 ratio using HPLC mobile phase (0.1-M sodium phosphate buffer/acetonitrile, 90:10, pH 3.0) prior to injection. Sample solutions (0.08 mg/ml; 20 μL) were injected into the HPLC system and analyzed using a UV/Vis detector (Waters 2695 Alliance System, USA). Separation was performed on a Waters Symmetry C_18_ Column (4.6 mm × 75 mm × 3.5 μm) at the column temperature of 30°C and UV detection wavelength of 254 nm. The mobile phase was pumped isocratically at a flow rate of 1.5 ml/min.

**Table II:** Fasted State Dissolution Testing in USP Apparatus III

Vessel	Media (pH)	Residence time (min)	Sampling time (min)
1	FaSSGF (1.8)	60	60
2	FaSSIF (6.5)	15	75
3	FaSSIF (6.8)	15	90
4	FaSSIF (7.2/halved bile salts)	30	120
5	Blank FaSSIF (7.5)	120	240
6	Blank FaSSIF (6.5)	720 (pull every 120)	360, 480, 600, 720, 840, 960

**Table III:** Fed State Dissolution Testing in USP Apparatus III

Vessel	Media (pH)	Residence time (min)	Sampling time (min)
1	Ensure® plus (6.4)	120	120
2	FeSSIF (5.0)	45	165
3	FeSSIF (6.5)	45	210
4	FeSSIF (6.5/halved bile salts)	45	255
5	Blank FaSSIF (7.5)	45	300
6	Blank FaSSIF (6.5)	720 (pull every 120)	420, 540, 660, 780, 900, 1020

### Stability Study

The HCTZ matrix tablets coated with Surelease and Opadry pore former were subjected to stability study. The tablets were packaged in HDPE bottles with desiccant, heat sealed, and stored at 30°C/65% RH and 40°C/75% RH for 6 months. The tablets were evaluated at 0, 3, and 6 months for physical properties, assay, and drug release.

## RESULTS AND DISCUSSION

**Fig. 2:**
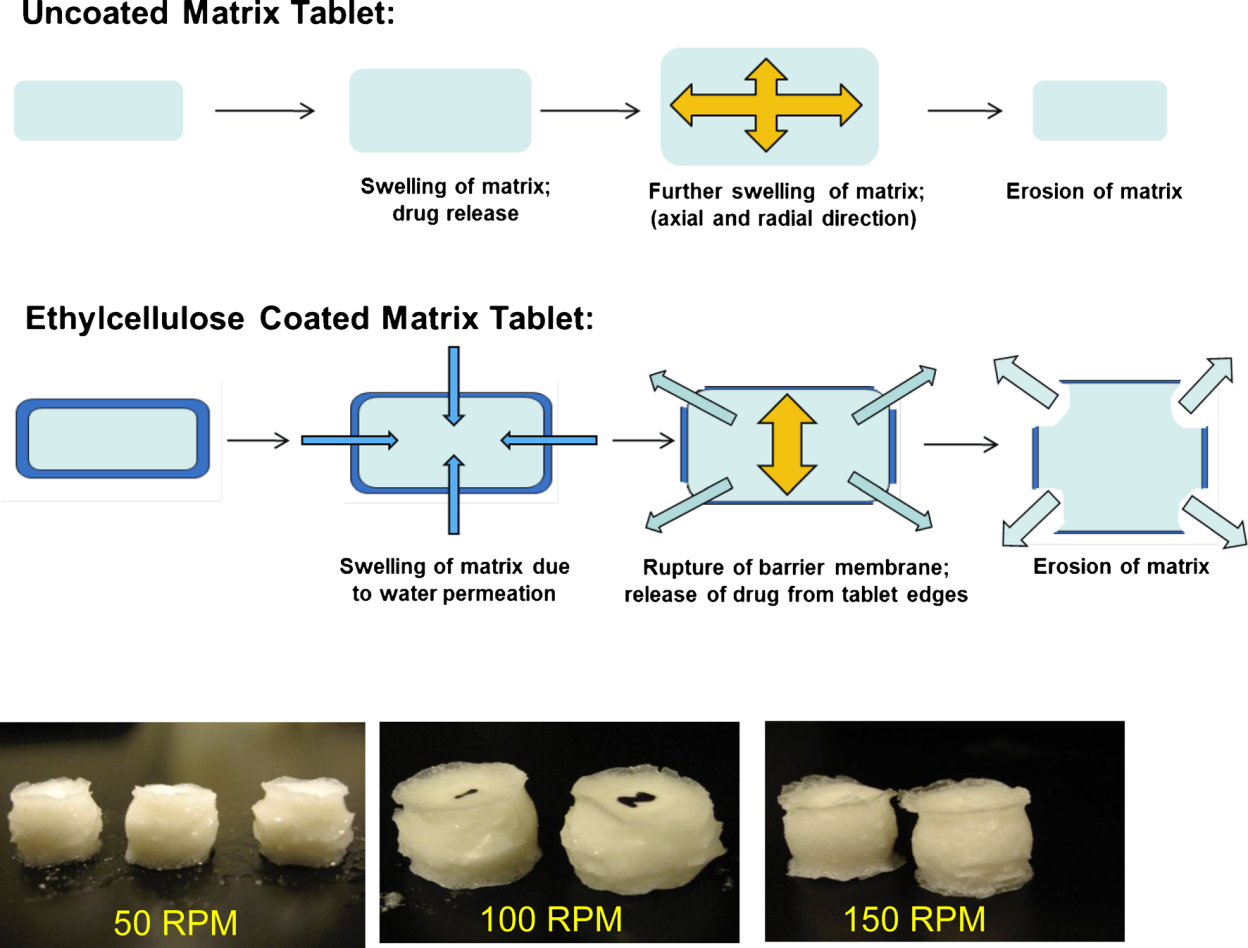
Fate of uncoated and aqueous ethylcellulose coated matrices in dissolution media (tablet images show ethylcellulose coated matrix tablets)

The fate of uncoated and barrier membrane-coated matrix tablets upon exposure to dissolution media is shown in Fig. 2. Upon media uptake, both systems exhibit hydration, swelling, and gel formation followed by tablet erosion. For uncoated matrix tablets, drug release starts to occur from all tablet surfaces upon hydration of tablet peripheries. For ethylcellulose coated tablets, the media permeates through the barrier membrane, results in increased hydrostatic pressure within the system which ultimately leads to the rupture of coating on tablet edges (belly band area). The rupture of barrier membrane occurs in a controlled and consistent fashion, providing only the belly band as the preferential area available for drug release. In general, a lag phase (0.5–2 h) is observed for drug release profiles from barrier membrane-coated matrices which is associated with the time required for media uptake into the tablets and coating rupture. The noted difference in hydration subsequently leads to a distinct difference in drug release profiles for uncoated and ethylcellulose coated matrix tablets, as discussed in the following sections.

### Drug Release from Uncoated Matrix Tablets

**Fig. 3:**
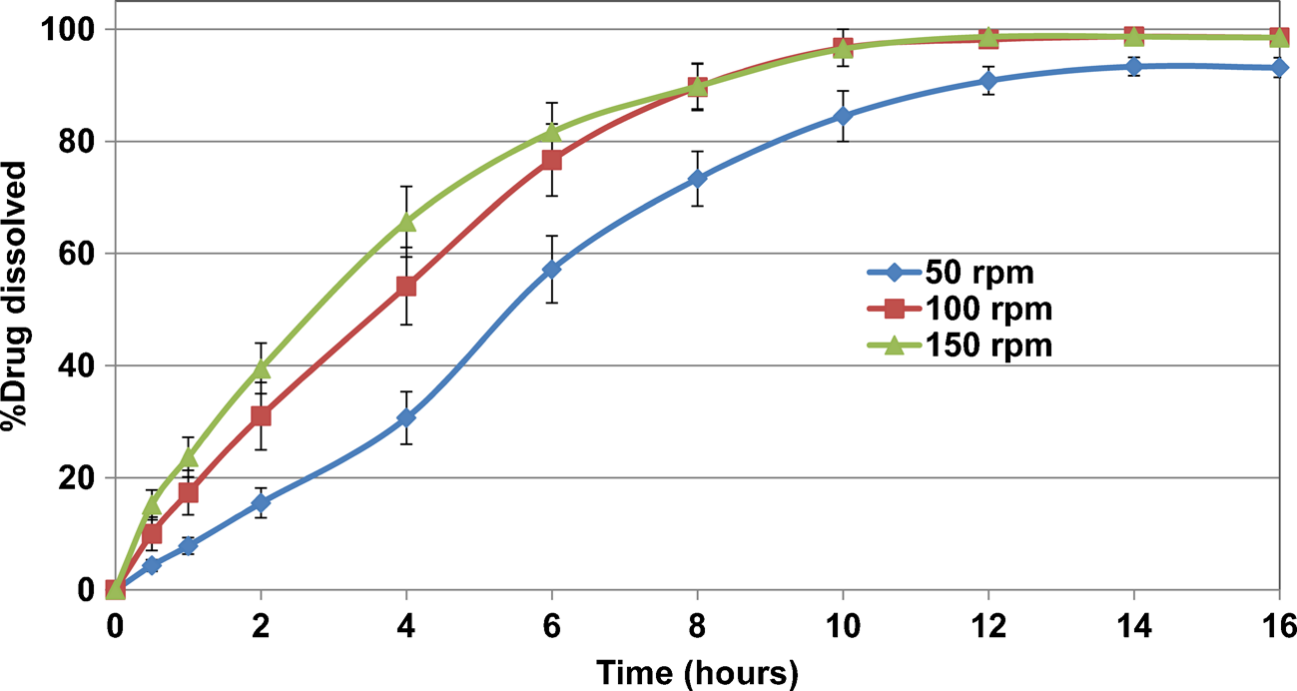
Drug release profiles of uncoated HCTZ matrix tablets containing lactose, as filler, in compendial media at different agitation rates

**Fig. 4:**
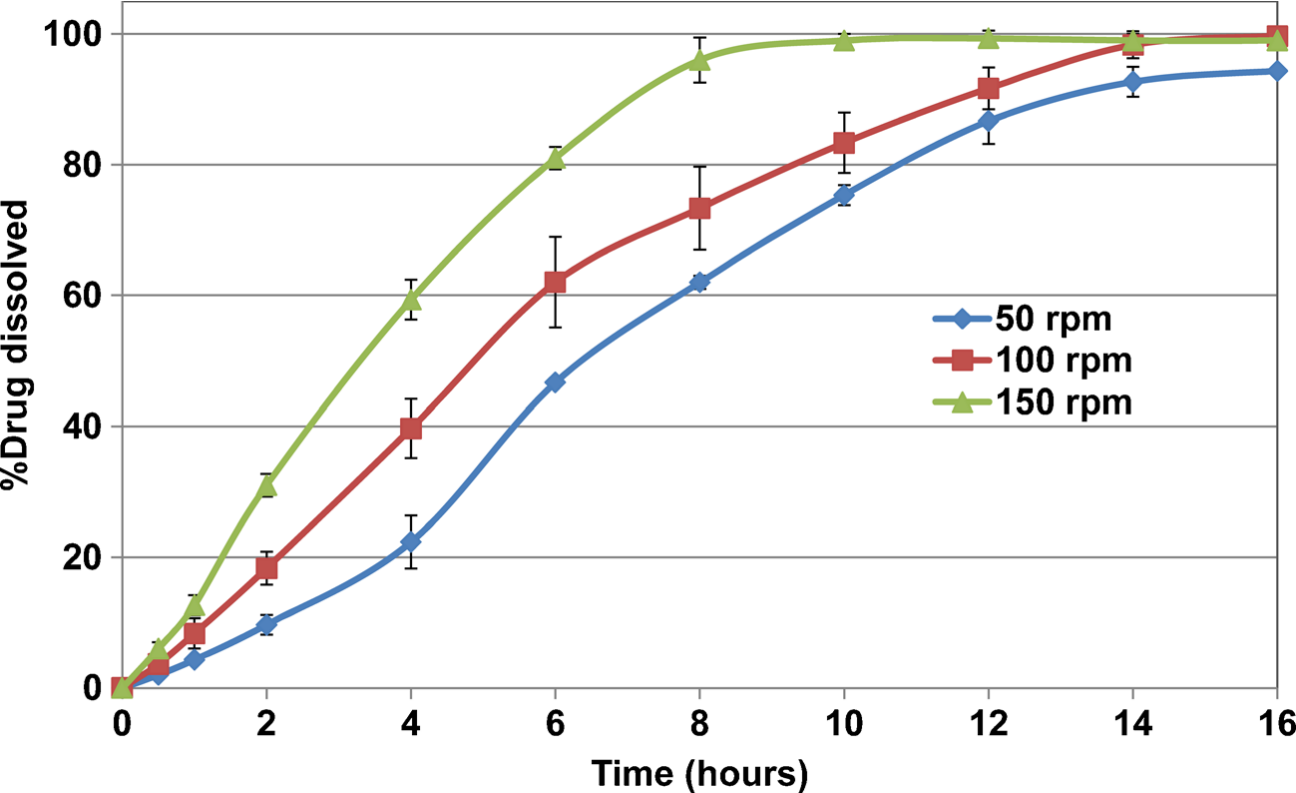
Drug release profiles of uncoated HCTZ matrix tablets containing Starch 1500, as filler, in compendial media at different agitation rates

Drug release from uncoated HCTZ matrix tablets containing lactose and Starch 1500, as fillers, in compendial media are illustrated in Figs. 3 and 4. The data points represent the mean value for the evaluated tablets and the error bars signify the standard deviation. The results revealed that drug release from uncoated matrices showed sensitivity to the agitation rates, ranging from 50 to 150 rpm. As the agitation rate was increased in the dissolution system, the drug release rate from matrices also increased. Such *in vitro* behavior may indicate variable *in vivo* release rate and potential post-prandial effect. Even though the same effect was observed for all uncoated matrices, the tablets containing Starch 1500 showed lower values of standard error bars compared to those containing lactose, indicating more robust drug release at any given agitation rate.

### Drug Release from Ethylcellulose Coated Matrix Tablets

**Fig. 5:**
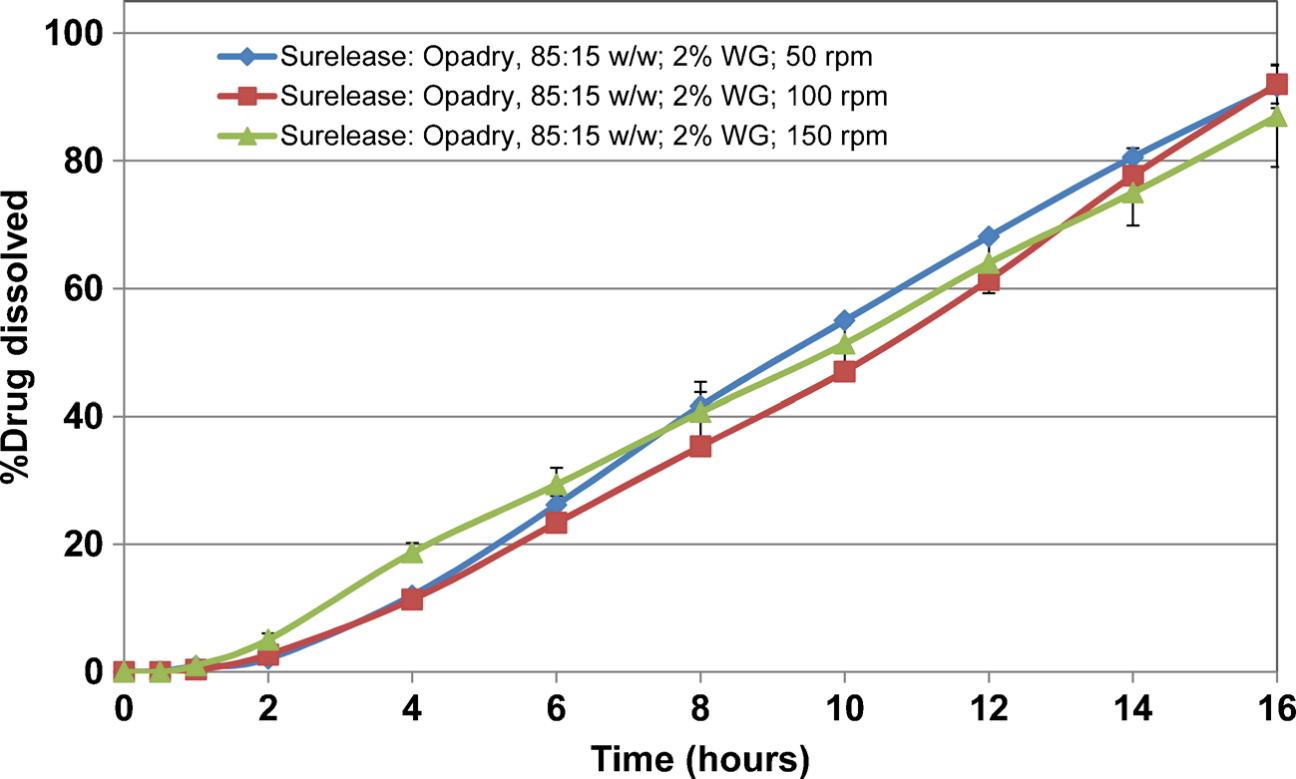
Drug release profiles of aqueous ethylcellulose coated HCTZ matrix tablets containing lactose in compendial media (coated with Surelease/Opadry, 85:15 *w/w*, 2% *w/w* weight gain (WG))

**Fig. 6:**
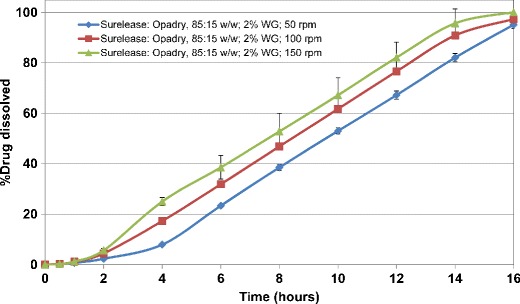
Drug release profiles of aqueous ethylcellulose coated HCTZ matrix tablets containing Starch 1500 in compendial media (coated with Surelease/Opadry, 85:15 *w/w*, 2% *w/w* weight gain (WG))

The drug release from ethylcellulose coated matrix tablets was also evaluated in a similar manner. Figures 5 and 6 show the release profiles for HCTZ matrices coated with aqueous ethylcellulose (Surelease/Opadry, 85:15 *w/w*) at 2% *w/w* weight gain. All matrices showed lag time of approximately 2 h followed by linear release. The results showed that ethylcellulose coating of HCTZ matrix tablets is effective in reducing the variability of drug release from the tablets. Application of higher weight gains of aqueous ethylcellulose coating (i.e., more than 2% *w/w*) resulted in longer lag time (between 2 and 4 h) as well as incomplete terminal drug release from all matrix tablets and hence is not recommended.

**Fig. 7:**
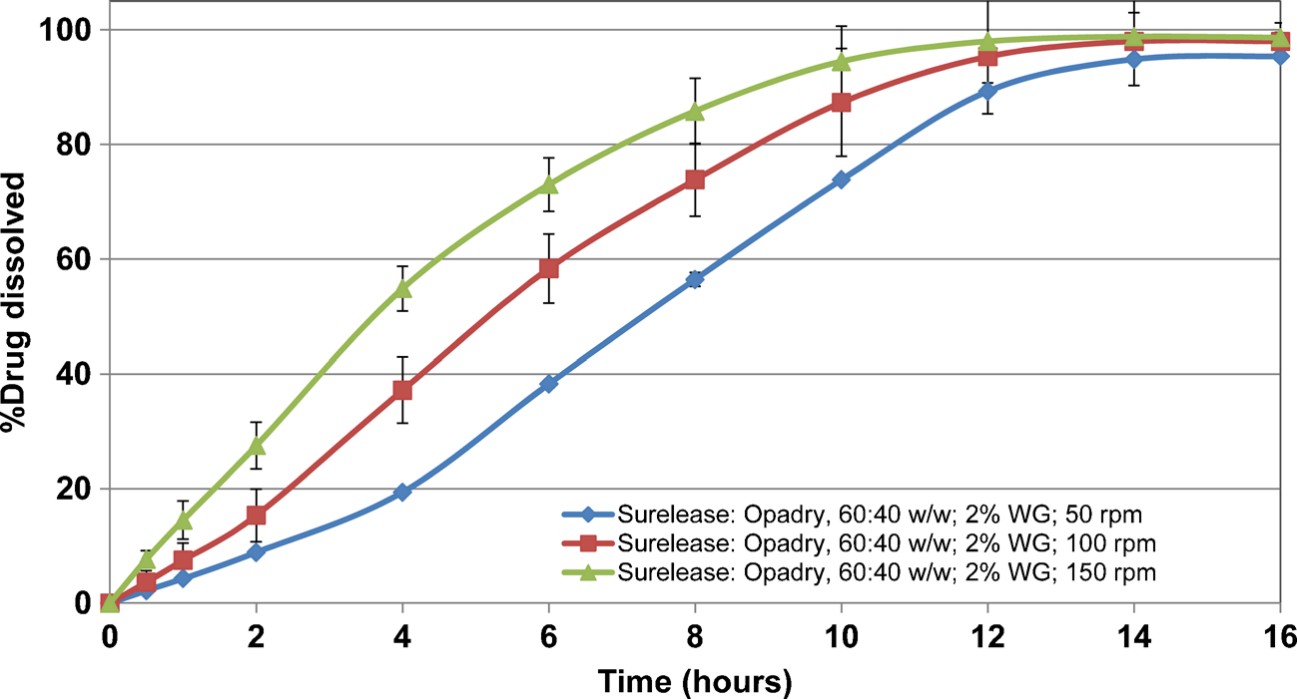
Drug release profiles of aqueous ethylcellulose coated HCTZ matrix tablets containing lactose in compendial media (coated with Surelease/Opadry, 60:40 *w/w*, 2% *w/w* weight gain (WG))

**Fig. 8:**
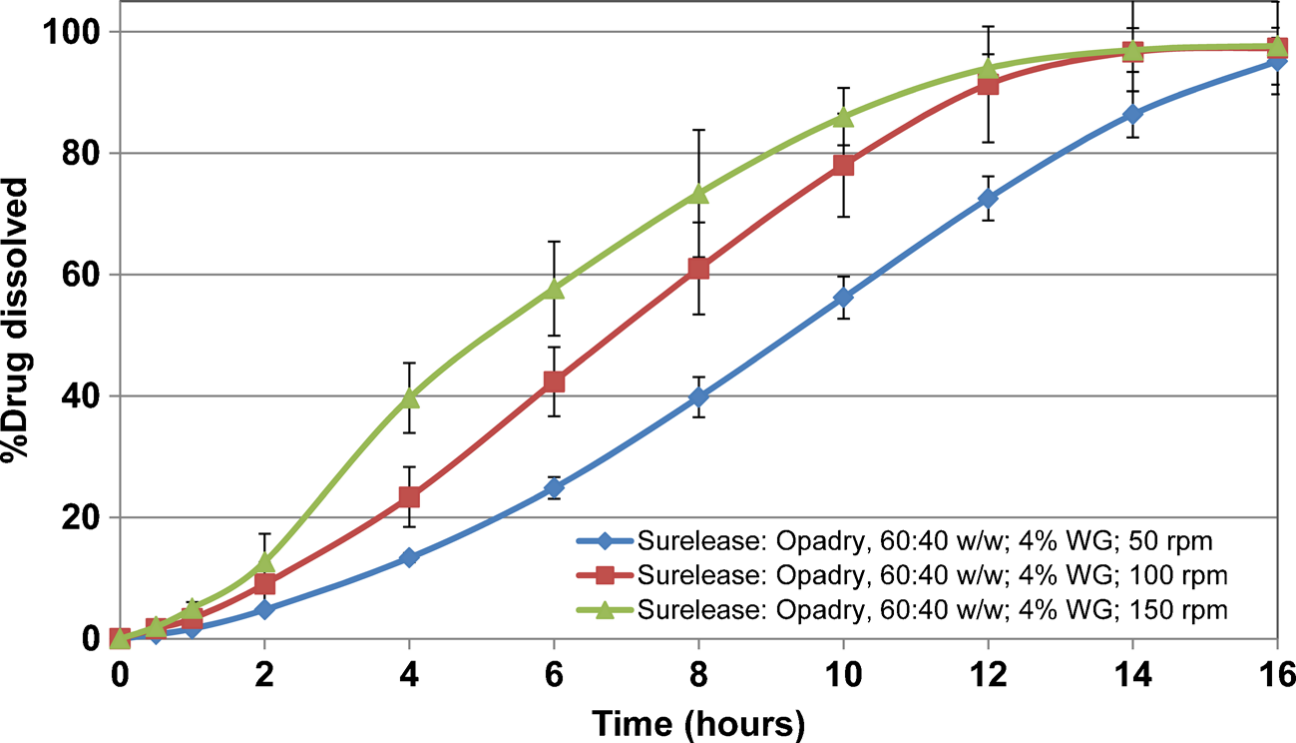
Drug release profiles of aqueous ethylcellulose coated HCTZ matrix tablets containing lactose in compendial media (coated with Surelease/Opadry, 60:40 *w/w*, 4% *w/w* weight gain (WG))

In order to evaluate the effect of pore former (Opadry) content, the HCTZ matrix tablets containing lactose were coated with Surelease/Opadry combination at 60:40 *w/w* at 2% and 4% *w/w* weight gain. The results showed that increasing the pore former content from 15% to 40% *w/w* within the ethylcellulose coating resulted in elimination of lag time followed by first-order drug release kinetics. This behavior was more evident at the lower coating weight gain (2% *vs.* 4% *w/w*) (Figs. 7 and 8). In addition, the higher level of pore former resulted in more variability in drug release upon increasing the agitation rate. Therefore, based on the obtained results, the ethylcellulose barrier membrane with the pore former content of 15% *w/w* was selected as the preferred system.

### Drug Release Mechanism for Uncoated and Ethylcellulose Coated Matrix Tablets

Drug release was characterized and the performance of matrix tablets were compared using the three-point dissolution data (*t*_10%_, *t*_50%_, *t*_90%_, time required to release 10%, 50%, and 90% of the drug), at different agitation rates, for uncoated and ethylcellulose coated matrices (Tables IV and V). In order to analyze the drug release mechanism, for uncoated matrix tablets, the dissolution data (∼5–85%) were fitted to the Power Law Model (17,18). For ethylcellulose coated matrices, the drug release profiles in the range of 5–95% were linear and accordingly the dissolution data provided a suitable fit to zero-order release kinetics. In all instances, the correlation coefficients (*R* ^2^) for the data were equal to or greater than 0.99.

**Table IV:** Comparison of Drug Release Rate and 3-Point Dissolution Data for Uncoated HCTZ Matrix Tablets at Different Agitation Rates (Power Law model), (a) Starch 1500 Formulation; (b) Lactose Formulation

	50 rpm	100 rpm	150 rpm
(a) Uncoated matrix - Starch 1500 formulation			
*k* (Kinetic constant)	4.4	8.5	13.4
*n* (Diffusional exponent)	1.25	1.04	1.03
*R* ^2^ (Correlation coefficient)	0.9955	0.9940	0.9913
*t*_10%_ (h)	2.0	1.2	0.8
*t*_50%_ (h)	6.4	4.8	3.3
*t*_90%_ (h)	13.0	11.6	7.0
(b) Uncoated matrix - lactose formulation			
*k* (Kinetic constant)	8.2	17.8	24.4
*n* (Diffusional exponent)	1.01	0.75	0.64
*R* ^2^ (Correlation coefficient)	0.9944	0.9900	0.9910
*t*_10%_ (h)	0.6	0.2	0.2
*t*_50%_ (h)	5.4	3.6	2.8
*t*_90%_ (h)	12.0	8.0	8.0

**Table V:** Comparison of Drug Release Rate and 3-Point Dissolution Data for Ethylcellulose Coated HCTZ Matrix Tablets at Different Agitation Rates (Zero-Order Kinetics/Linear Equation), (a) Starch 1500 Formulation; (b) Lactose Formulation

	50 rpm	100 rpm	150 rpm
(a) Coated matrix - Starch 1500 formulation			
*k* (Release rate, %/h)	7.3	7.3	7.3
*R* ^2^ (Correlation coefficient)	0.9983	0.9983	0.9971
*t*_10%_ (h)	4.4	3.0	2.6
*t*_50%_ (h)	9.6	8.4	7.6
*t*_90%_ (h)	15.0	14.0	13.0
(b) Coated matrix - lactose formulation			
*k* (Release rate, %/h)	6.6	6.4	5.8
*R* ^2^ (Correlation coefficient)	0.9985	0.9947	0.9994
*t*_10%_ (h)	3.6	3.8	2.8
*t*_50%_ (h)	9.2	9.8	10.4
*t*_90%_ (h)	15.8	15.8	16.2

For uncoated matrix tablets containing Starch 1500, increasing the agitation rate resulted in an increase in kinetic constant (*k*, indicative of drug release rate), in the order of 4.4, 8.5, and 13.4 for 50, 100, and 150 rpm, respectively. The uncoated matrices comprising lactose demonstrated faster release rates of 8.2, 17.8, and 24.4, confirming that the use of Starch 1500 as a filler within the HPMC matrix tablets of HCTZ led to more extended drug release under similar testing conditions. Additionally, comparing the values for *t*_10%_, *t*_50%_, and *t*_90%_ among the matrix tablets confirmed the above results (Table IV). The values of the release exponent (*n,* indicative of drug release mechanism) were in the range of 1.03–1.25 and 0.64–1.01 for matrix tablets containing Starch 1500 or lactose, as fillers, respectively. For cylindrical matrices, Fickian diffusion is represented by *n* = 0.45; for non-Fickian release (anomalous behavior), 0.45 < *n* < 0.89; for Case II transport, *n* = 0.89; and for Super-Case II transport, *n* > 0.89 (17–19). Accordingly, the obtained results revealed a Super-Case II transport for matrix tablets containing Starch 1500 where polymer relaxation is the main contributor to the drug release. In such cases, the release rate accelerates at later stages leading to a more rapid relaxation-controlled transport (20,21). For matrices containing lactose, drug release is governed by a combination of diffusion and erosion, except for when these tablets are subjected to the lower agitation rate of 50 rpm for which the drug release mechanism demonstrates a Super-Case II transport.

For ethylcellulose coated matrix tablets, the release rates (*k*) at varying agitation speeds were calculated using the linear section of the dissolution profiles (Table V). The results for all coated tablets exhibited less sensitivity to the agitation speed, as evidenced from the drug release rates values of 7.3%/h for the tablets containing Starch 1500, and 6.6–5.8%/h when lactose was used as the filler. The calculated values of *t*_10–90%_ were also in agreement with the release rate values, demonstrating less variation compared to the uncoated matrix tablets.

### Drug Release in Bio-Relevant Media

**Fig. 9:**
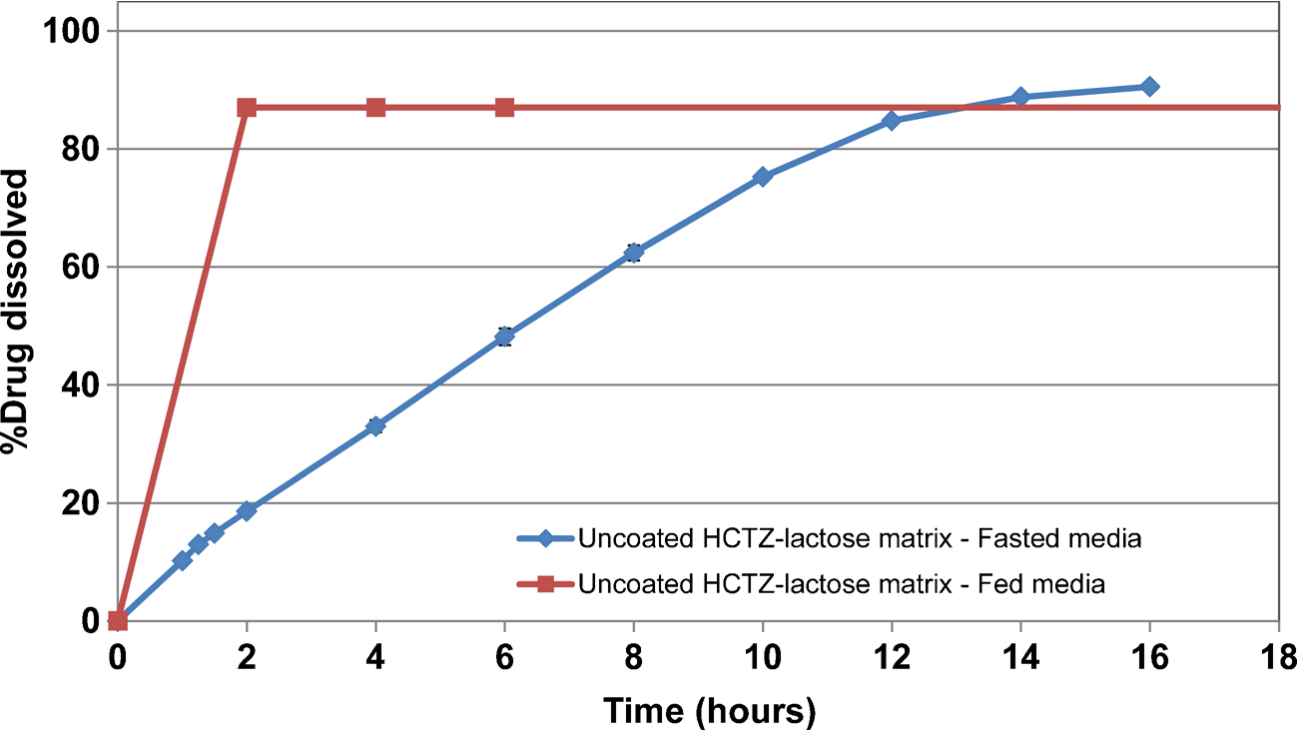
Dissolution profiles for uncoated HCTZ matrices containing lactose, as filler, in bio-relevant fasted and fed media

Furthermore, the HCTZ matrix tablets, uncoated as well as coated with the preferred ethylcellulose coating system (Surelease/Opadry, 85:15 *w/w*), were subjected to dissolution testing in bio-relevant media. As shown in Fig. 9, the drug release from uncoated matrix tablets containing lactose was in a controlled release fashion in simulated fasted media (FaSSIF), while dose dumping was observed under fed conditions (FeSSIF). The faster drug release rate was due to disintegration and rapid erosion of uncoated matrix tablets during exposure to the first phase of the fed media. This presented the challenge of the sink-limiting condition for quantification of the actual amount of the released drug. Further dilution and stirring was used for accurate analysis of the drug.

**Fig. 10:**
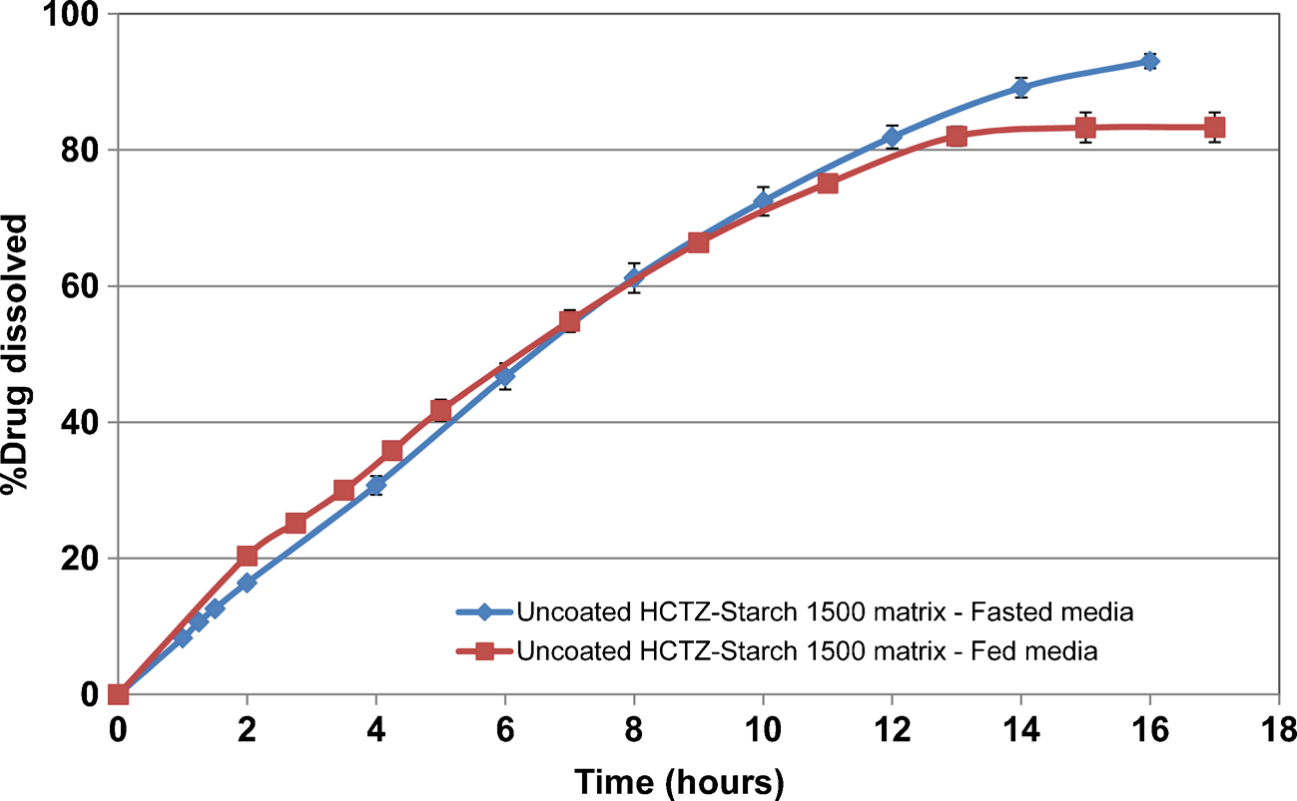
Dissolution profiles for uncoated HCTZ matrices containing Starch 1500, as filler, in bio-relevant fasted and fed media

**Fig. 11:**
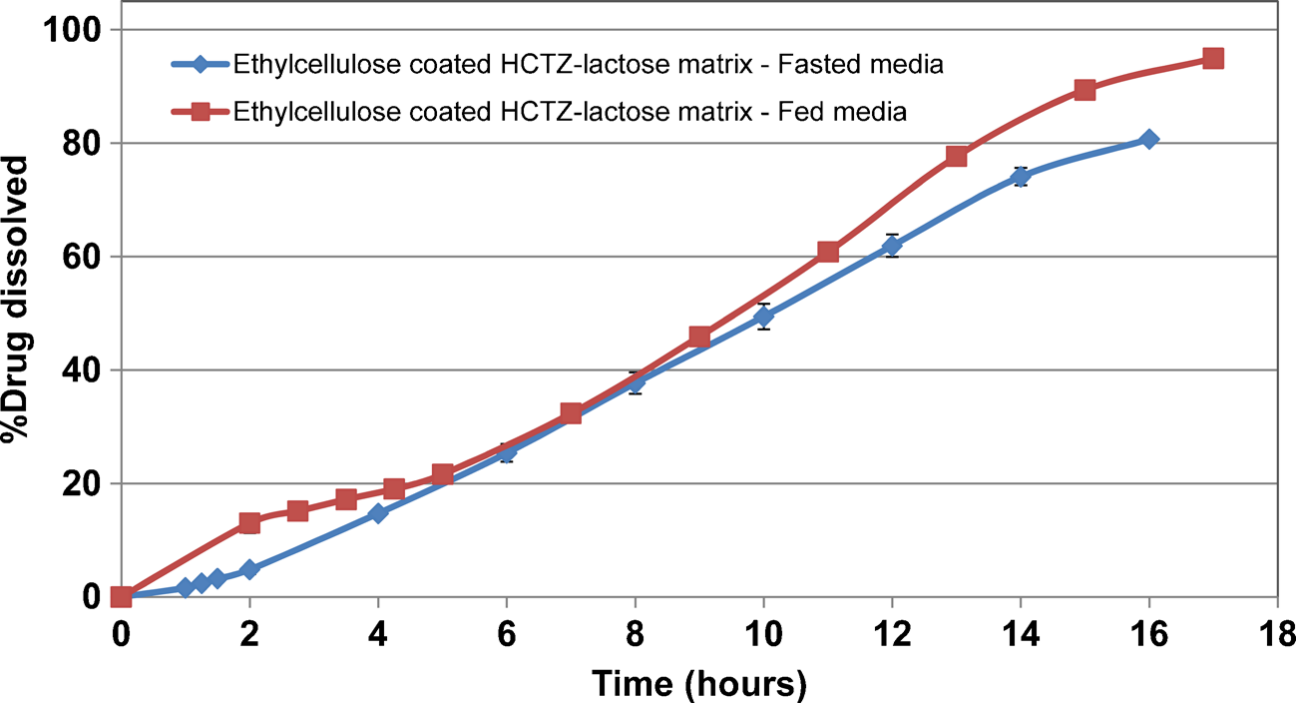
Dissolution profiles for aqueous ethylcellulose coated HCTZ matrices containing lactose in bio-relevant fasted and fed media (coated with Surelease/Opadry, 85:15 *w/w*, 2% *w/w* weight gain (WG))

In comparison, the inclusion of Starch 1500 within the uncoated matrix tablets minimized rapid disintegration and erosion of the uncoated HCTZ matrices and resulted in greater extended drug release which was similar in fasted and fed-simulated media (Fig. 10). This effect could be due to the contribution of Starch 1500 to formation of a stronger gel layer around the matrix tablet (14,22). Application of aqueous ethylcellulose coating resulted in more robust drug release profiles in both fasted and fed media, irrespective of the filler type. The release profiles for HCTZ matrices containing lactose are shown in Fig. 11. Therefore, the application of barrier membrane coating of aqueous ethylcellulose dispersion along with an HPMC-based Opadry system, as a pore former, at 85:15 *w/w* ratio, and at 2% weight gain resulted in significant reduction in variability of release and provided zero-order release kinetics. The similarity factors, ƒ_2_ (23), were greater than 60 for all evaluated agitation rates. It was hypothesized that the consistent and uniform breakage of the film coating around the tablet edges was the reason for achieving robust drug release at various agitation rates. The fate of a HCTZ matrix tablet coated with Surelease–Opadry combination over time within the dissolution media is captured as a video (refer to Frame still), demonstrating matrix hydration and swelling followed by rupture of coating layer at the edges of the HCTZ tablet.

### Comparative Evaluation of Organic Ethylcellulose Coating

**Fig. 12:**
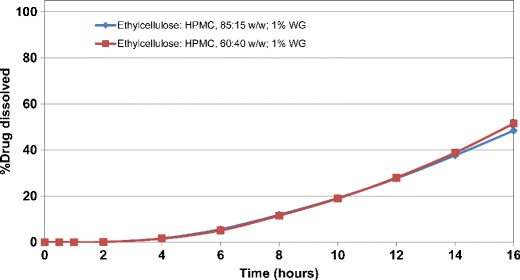
Drug release profiles of solvent-based ethylcellulose coated HCTZ matrix tablets containing lactose (coated with ETHOCEL 20/METHOCEL E5LV, 1% *w/w* weight gain (WG))

In comparison, application of solvent-based coating of ETHOCEL 20/METHOCEL E5LV, at 85:15 and 60:40 *w/w*, on HCTZ matrix tablets at 1% *w/w* weight gain resulted in longer lag time (∼5 h) followed by incomplete drug release (∼50% at 16 h) (Fig. 12). Increasing the pore former level from 15% to 40% *w/w* in the organic coating composition did not enhance the drug release from such matrices. Increasing the coating weight gain to 2% *w/w* completely shut down the drug release (data not shown). Matrix tablets showed swelling after 16 h of dissolution testing with no sign of coating rupture. The difference in performance of aqueous *versus* organic ethylcellulose coated matrices could be attributed to the nature of the film layer. The ethylcellulose coating, when applied organically, can accommodate the swelling matrix to a greater extent compared to aqueous coating and hence, coating rupture and subsequent drug release is prevented. This further confirms the suitability of aqueous ethylcellulose coating of matrix tablets as a preferred barrier membrane coating system, since it provides a consistent rupture pattern at the tablet edges upon exposure to the surrounding media.

### Stability Study

**Fig. 13:**
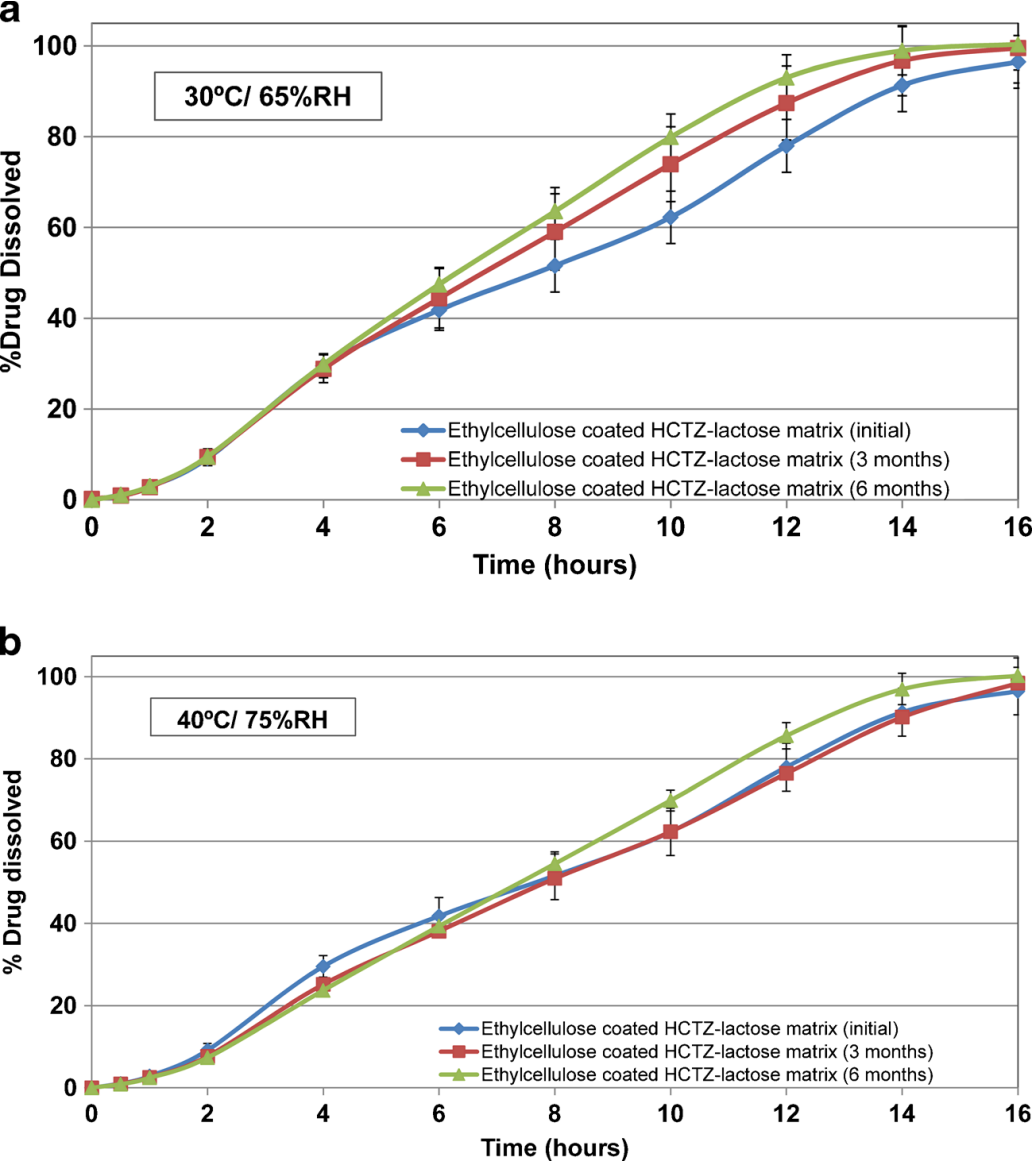
Drug release profiles of HCTZ matrix tablets containing lactose, as filler, and aqueous ethylcellulose coating (Surelease/Opadry, 85:15 *w/w,* 2% *w/w* weight gain (WG)) after storage in different conditions for 6 months, (**a**) 30°C/65%; (**b**) 40°C/75%

**Fig. 14:**
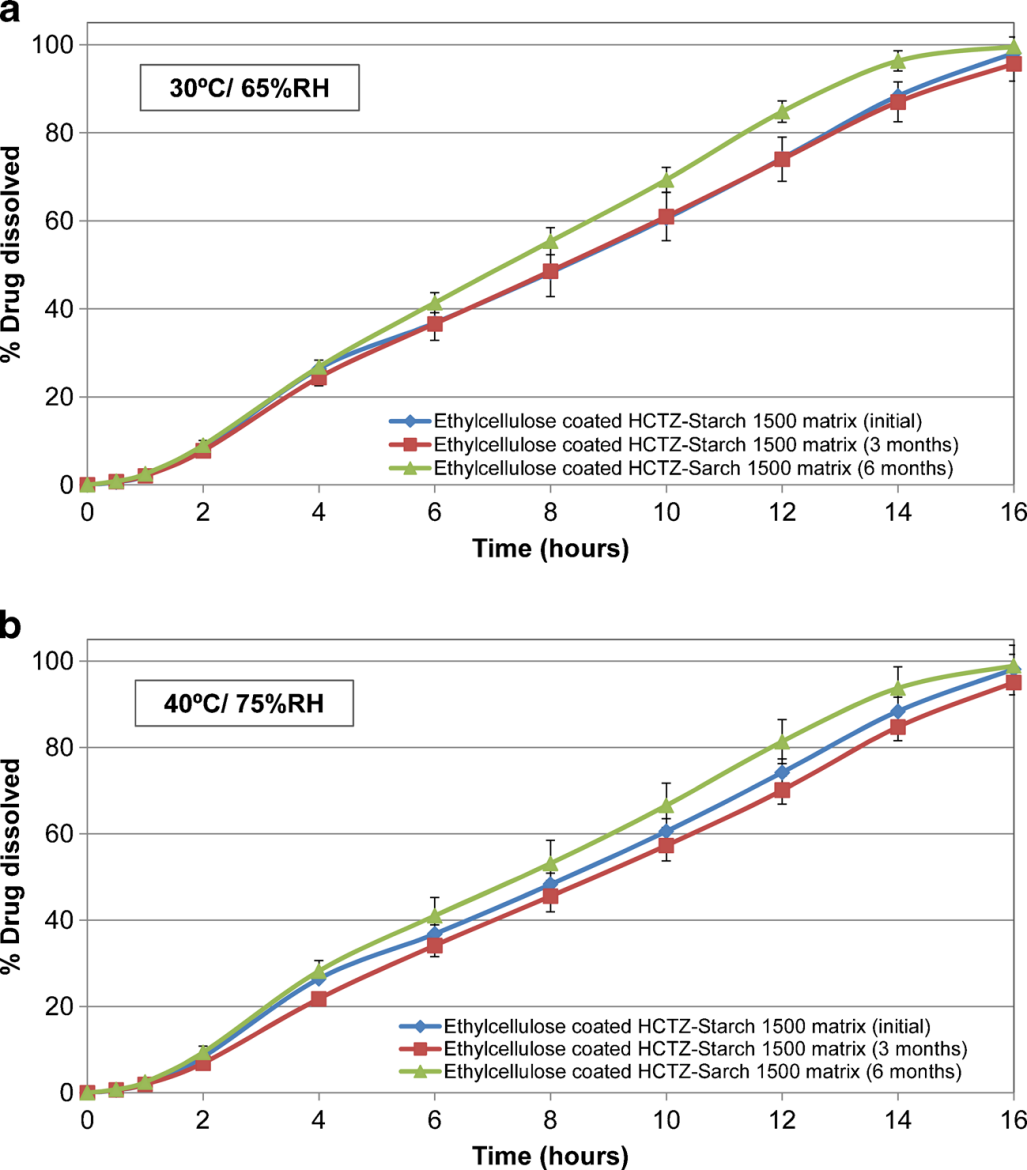
Drug release profiles of HCTZ matrix tablets containing Starch 1500, as filler, and aqueous ethylcellulose coating (Surelease/Opadry, 85:15 *w/w*, 2% *w/w* weight gain (WG)) after storage in different conditions for 6 months, (**a**) 30°C/65%; (**b**) 40°C/75%

Figures 13 and 14 exhibit the drug release from barrier membrane-coated matrix tablets at the initial time point, as well as upon storage at 30°C/65% RH and 40°C/75% RH conditions, respectively. Aqueous ethylcellulose coated matrix tablets containing lactose or Starch 1500 resulted in similar drug release under exposure to the stability conditions (Table VI). However, the matrices formulated with lactose (soluble filler) resulted in higher variability of drug release as evident from the greater standard error bars in Fig. 13 (average value = 3.9%; range = 0–10.2%). Inclusion of Starch 1500 (partially soluble filler) in formulation of HCTZ matrix tablets led to reduction of variability in drug release as evident from the smaller error bars in Fig. 14 (average value = 3.1%, range = 0–7.8%). This observation was further confirmed by comparison of *f*_*2*_ values (Table VI), where ethylcellulose coated matrices containing Starch 1500 demonstrated greater *f*_*2*_ values compared to the tablets containing lactose. This indicates that inclusion of Starch 1500 as filler results in more robust drug release from the hydrophilic matrices. The drug release profiles from the tablets, containing either filler, remained similar after storage for 6 months, when compared to their respective dissolution profiles obtained at the initial time point (*f*_*2*_ > 50 in both cases; Table VI).

**Table VI:** Comparison of Similarity Factors (*f*_*2*_) for Aqueous Ethylcellulose Coated HCTZ Matrix Tablets

Stability condition	Duration	Starch 1500	Lactose
40°C/75% RH	3 months	85	77
	6 months	65	64
30°C/65% RH	3 months	94	60
	6 months	63	52

Release profiles for each filler at initial time point was considered as reference

## CONCLUSIONS

The application of a barrier membrane coating system consisting of aqueous ethylcellulose dispersion (Surelease) and an HPMC-based Opadry system, as a pore former, was found to be a promising strategy to obtain robust and consistent drug release profiles from hydrophilic matrix tablets of hydrochlorothiazide. The coated matrices showed minimal sensitivity to varying agitation rates and simulated post-prandial effects. The barrier membrane weight gain and inclusion level of the pore former within the coating are critical in achieving consistent drug release profiles which may further resist highly variable mechanical forces acting on matrix formulations in fed and fasted states. Aqueous ethylcellulose coated matrices, with either Starch 1500 or lactose as fillers, provided consistent and stable drug release profiles, irrespective of the storage conditions. Use of Starch 1500, in particular, contributed to enhanced robustness of the matrix tablets. Such a formulation strategy may provide options for the development of dosage forms where zero-order drug release kinetics is desired. This study, therefore, highlighted various approaches which could be used either alone or in combination to successfully formulate robust hydrophilic matrices of a low-soluble drug with minimal susceptibility to drug release variability.

## Electronic supplementary material

Below is the link to the electronic supplementary material.

ESM 1 Frame still (video). Fate of barrier membrane-coated HCTZ matrix tablets containing lactose, as filler, and aqueous ethylcellulose coating (Surelease/Opadry, 85:15 *w/w*, 2% *w/w* weight gain (WG)) in dissolution media over time (WMV 2489 kb)
